# *Achyranthes bidentata* Polysaccharide Activates Nuclear Factor-Kappa B and Promotes Cytokine Production in J774A.1 Cells Through TLR4/MyD88 Signaling Pathway

**DOI:** 10.3389/fphar.2021.753599

**Published:** 2021-10-01

**Authors:** Sairong Fan, Yanxing Wang, Yue Zhang, Yamin Wu, Xiaoming Chen

**Affiliations:** ^1^ Institute of Glycobiological Engineering, School of Laboratory Medicine and Life Sciences, Wenzhou Medical University, Wenzhou, China; ^2^ Zhejiang Provincial Key Laboratory of Medical Genetics, Key Laboratory of Laboratory Medicine, Ministry of Education, School of Laboratory Medicine and Life Sciences, Wenzhou Medical University, Wenzhou, China

**Keywords:** *Achyranthes bidentata*, polysaccharides, immunomodulatory, Toll-like receptors (TLRs), NF-kappa B

## Abstract

*Achyranthes bidentata* Blume, a traditional Chinese medicine, is widely acknowledged for its function of invigorating the liver and kidneys and as a stranguria-relieving diuretic and used in the treatment of edema, gonorrhea, and other diseases. Polysaccharide (ABPS), isolated from *Achyranthes bidentata* Blume, has been demonstrated to have multiple biological activities including immunomodulatory effects. However, the mechanisms underlying the effects of ABPS have not been fully investigated. The present study is conducted to explore the underlying mechanism of immunomodulatory activities of ABPS. Results showed that ABPS significantly increased the secretion of IL-1β and TNF-α in J744 A.1 cells. Nitric oxide (NO) also significantly increased after ABPS treatment. The special antibodies (Toll-like receptor 4 (TLR4) antibody and CD14/TLR4 antibody) significantly decreased the activation, while the Toll-like receptor 2 (TLR2) antibody could not abolish this activation. Meanwhile, pyrrolidine dithiocarbamate (PDTC), a specific inhibitor of NF-κB, remarkably inhibited the secretion of IL-1β and TNF-α induced by ABPS in J744 A.1 cells. Western blotting (WB) and confocal laser scanning microscopy (CLSM) showed that ABPS promoted NF-κB translocation into the nucleus. Furthermore, the mRNA and protein expression of TLR4 and MyD88 were significantly increased after ABPS treatment. Taken together, these findings suggested that the immunomodulatory mechanism of ABPS was associated with the secretion of cytokines by stimulating the NF-κB pathway through TLR4/MyD88 signaling.

## Introduction

Polysaccharides, isolated from various natural sources, possess diverse biological and pharmacological activities including immunomodulatory, anti-inflammatory, antioxidant, antidiabetic, antimutagenic, antiviral, and antitumor (Guo et al., 2019; Shalini et al., 2019; Mohammed et al., 2021). Notably, numerous studies have shown that polysaccharides, especially from traditional Chinese medicine, exerted significant immune regulation activities and widely applied in pharmaceutical fields (Li C. et al., 2021; Wang et al., 2021).

*Achyranthes bidentata* Blume (named Huai Niu Xi, a member of the Amaranthaceae family), a traditional Chinese medicinal herb, is widely used in China, Korea, and Japan for its function of nourishing the kidney and liver and strengthening muscles and bones (Ou et al., 2018; Ao and Kim, 2020). The polysaccharide (ABPS) is extracted from *A. bidentata*. Pharmacological studies have demonstrated that ABPS has multiple functions, such as antiosteoporosis, anti-inflammatory, antioxidant, antiallergic, and immunomodulatory (Yan et al., 2019; Ao and Kim, 2020). However, the mechanism of the immunomodulatory activity of ABPS is unclear.

It has been known that polysaccharides can directly or indirectly activate macrophages, natural killer cells, T lymphocytes, dendritic cells, etc., increase the secretion of cytokines, improve the antibody level, and enhance the immune response through different mechanisms (Yu et al., 2018; Chen et al., 2019; Li Y. et al., 2021). The innate immune response relies on several germ line–encoded pattern-recognition receptors (PRRs) (Ahmed et al., 2021). Toll-like receptors (TLRs), as one of the largest and well-characterized PRRs, have a key role in the immune response (El-Zayat et al., 2019; Mokhtari et al., 2021). Upon stimulation, TLRs bind to the adaptor protein and trigger downstream signaling cascades, which lead to the activation and nuclear translocation of the transcription factors such as interferon regulatory factors (IRFs) and nuclear factor-kappa B (NF-κB) (Mokhtari et al., 2021; Seumen et al., 2021). The NF-κB signaling leads to release the inflammatory cytokines, such as interleukin-1β (IL-1β), interleukin-6 (IL-6), and tumor necrosis factor α (TNF-α) (Liu et al., 2017; Denning et al., 2019). Consequently, the TLR-initiated signaling cascades activate the immune responses (Fitzgerald and Kagan, 2020).

This study was designed to investigate the immunomodulatory effect of ABPS and the underlying molecular mechanism. The results indicate that ABPS promotes the secretion of cytokines and activates the immune response by stimulating the NF-κB pathway through TLR4/MyD88 signaling. This study provides a basis for the development and utilization of ABPS as an agonist of TLR4 in the future.

## Materials and Methods

### Materials and Chemicals

Lipopolysaccharide (LPS), pyrrolidine dithiocarbamate (PDTC), propidium iodide (PI), and fluorescein isothiocyanate (FITC) were purchased from Sigma Chemical Co. (MO, United States). Fetal bovine serum (FBS) and RPMI 1640 were purchased from Gibco (NY, United States). Cytokine (IL-1β and TNF-α)-detecting ELISA kits were purchased from Changfeng Biotechnology Co. (Zhejiang, China). The assay kit for nitric oxide (NO) was purchased from Jiancheng Biologic Project Company (Nanjing, China). Antibodies against Toll-like receptor 2 (TLR2), cluster of differentiation 14 (CD14), and TRIF-related adaptor molecule (TRAM) were purchased from Proteintech (IL, United States). A nuclear factor-kappa B (NF-κB) antibody was purchased from Santa Cruz Biotechnology Inc. (TX, United States). Antibodies against Toll-like receptor 4 (TLR4) and myeloid differentiation factor 88 (MyD88) were purchased from Bioworld Biotech Co. (Nanjing, China). TRIZOL reagent and cDNA reverse transcription kits were obtained from TaKaRa Biotechnology Co., Ltd. (Dalian, China).

### Preparation and Structural Characterization of ABPS

ABPS was prepared and characterized as previously reported (Chen et al., 2005) and kindly provided by Prof. Gengyuan Tian (Shanghai Institute of Organic Chemistry, Chinese Academy of Sciences). Briefly, the dried roots of *A. bidentata* were sliced into sheets and extracted with distilled water at room temperature overnight. The water extract was filtered and concentrated under vacuum, and supernatants were precipitated with acetone at 4°C overnight. The crude polysaccharides were deproteinized, filtered, concentrated, and loaded on a DEAE-Cellulose 52 column and Sephadex G-50 column to obtain ABPS. The homogeneity and molecular weight of ABPS were determined by high-performance gel permeation chromatography (HPGPC) with TSK-2000SW columns. The monosaccharide composition of ABPS was determined by high-performance liquid chromatography (HPLC) with a carbohydrate analysis column. Infrared spectral analysis was conducted on a Bio-Rad FTS185 spectrophotometer, and the ^13^C NMR spectra of ABPS were recorded with a Bruker AMX-600 spectrometer.

ABPS consisted of fructose and glucose in a molar ratio of 8:1, with a mean molecular weight of 1,400 Da. ABPS has a main chain of (2→1)-linked-β-D-Fruf and a branch chain of (2→6)-linked-β-D-Fruf with (2→1,6)-linked-β-D-Fruf residues and terminated with fructose and glucose residue (Chen et al., 2005).

### Cell Culture

J774 A.1 cell line (mouse monocyte/macrophage) was obtained from the Cell Resource Center, Chinese Academy of Sciences (Shanghai, China) and cultured in RPMI 1640 supplemented with 10% FBS at 37°C and 5% CO_2_.

### IL-1β and TNF-α Measurement by ELISA

J774 A.1 cells were seeded in a 24-well plate containing 1 × 10^6^ cells per well. The cell culture medium containing ABPS at various concentrations (50, 200, 500, or 1,000 μg/ml) was added to each well. Cells treated with 5 μg/ml LPS were set as the positive control, and cells treated with the medium only were set as the negative control. Following culture for 24 h, the culture supernatants were collected. The levels of IL-1β and TNF-α were detected using commercial ELISA kits in accordance with the manufacturer’s instructions. The absorbance was read at 450 nm on an automatic ELISA plate reader.

In order to observe the effect of ABPS on the activation of TLRs signaling, J774 A.1 cells (1 × 10^6^ cells/well) were pretreated with or without 20 μg/ml antibody (anti-TLR2, anti-TLR4, or anti-TLR4/CD14) for 1 h, and then, ABPS (0, 50, 200, 500, and 1,000 μg/ml) or LPS (5 μg/ml) was added into the cell supernatant. After cultured for 24 h, the culture supernatants were collected for the detection of IL-1β and TNF-α using commercial ELISA kits.

In order to observe the effect of ABPS on the activation of NF-κB, J774 A.1 cells (1 × 10^6^ cells/well) were pretreated with or without 100 μmol/L PDTC for 2 h, and then, ABPS (0, 50, 200, 500, and 1,000 μg/ml) or LPS (5 μg/ml) was added into the cell supernatant. After cultured for 24 h, the culture supernatants were collected for the detection of IL-1β and TNF-α.

### NO Measurement by the Colorimetric Method

J774 A.1 cells (1 × 10^6^ cells/well) were treated with ABPS (50, 200, 500, and 1,000 μg/ml). Cells treated with 5 μg/ml LPS were set as the positive control, and cells treated with the medium only were set as the negative control. Following culture for 24 h, the culture supernatants were collected for the detection of NO levels using a commercial kit at 550 nm and according to the manufacturer’s instructions.

### Reverse Transcription-Polymerase Chain Reaction

RT-PCR was performed as previously described (Chen et al., 2014). Total RNA was isolated using TRIZOL reagent according to the manufacturer’s protocol. The cDNA was synthesized using a commercially available cDNA reverse transcription kit in a Bio-Rad thermocycler. Polymerase chain reaction (PCR) amplification was performed using the following cycle: an initial denaturing at 94°C for 5 min, followed by 30 cycles of denaturing at 94°C for 30 s, annealing at 58°C (*GAPDH*) for 30 s, extension at 72°C for 1 min, and a final extension at 72°C for 10 min. The detailed information of specific primers is shown in Table 1. The relative mRNA level was calculated by normalization to GAPDH.

**TABLE 1:** Primer sequences for PCR.

Genes	Forward (5′–3′)	Reverse (5′–3′)	Annealing temperature (°C)
*GAPDH*	GAA​CAT​CAT​CCC​TGC​CTC​TAC​T	CCT​GCT​TCA​CCA​CCT​TCT​TG	58
*IL-1β*	TGA​TGA​CGA​CCT​GCT​AGT​GT	CTT​CTT​TGG​GTA​TTG​TTT​GG	53
*TNF-α*	CCA​CGC​TCT​TCT​GTC​TAC​TGA​ACT	GAG​GCT​GAC​TTT​CTC​CTG​GTA​TG	60.5
*TLR4*	ATC​GCA​TAG​AGA​CAT​CCA​AAG​G	GTTTCYCACCCAGTCCTCATTCT	57.2
*TLR2*	TCT​TGG​AAC​TGA​TGG​AGG​TGG​AG	ACA​ACT​GTC​GGG​CAT​AGG​CTG	60
*TRAM*	AGGAAAGCAGGAGGGAGC	AAG​GCA​TTG​ATG​GTT​TGG​AG	57
*MyD88*	TCCACATCCTCCCTTCCC	GAG​ACA​ACC​ACC​ACC​ATC​C	59

GAPDH, glyceraldehyde-3-phosphate dehydrogenase; IL-1β, interleukin 1 beta; MyD88, myeloid differentiation factor 88; TNF-α, tumor necrosis factor-α; TLR4, Toll-like receptor 4; TLR2, Toll-like receptor 2; TRAM, TRIF-related adaptor molecule.

### Western Blot

J774 A.1 cells were seeded in a 6-well plate containing 1 × 10^6^ cells per well. After incubated with or without ABPS (500 μg/ml) for 10 h, cells were washed with ice-cold PBS and lysed in radioimmunoprecipitation (RIPA) lysis buffer, and the protein of cell lysates was quantified by the BCA reagent. Harvested proteins were denatured at 100°C for 10 min, electrophoresed by sodium dodecyl sulfate polyacrylamide gel electrophoresis (SDS-PAGE), and then, transferred to polyvinylidene fluoride (PVDF) membranes (GE Healthcare, Silverwater, Australia). The membranes were preincubated for 1 h in PBS, 5% skim milk, and 0.1% Tween 20. After that, the membranes were incubated with primary antibody overnight at 4°C, following by incubation with horseradish peroxidase (HRP)–conjugated secondary antibodies. Finally, the bands were visualized with the enhanced chemiluminescence (ECL) system.

In order to observe the effect of ABPS on the activation of NF-κB, cells (1 × 10^6^ cells/well) were seeded in a 6-well plate. After treatment with various concentrations of ABPS (0, 50, 200, 500, and 1,000 μg/ml) or LPS (5 μg/ml) for 10 h, cells were washed with ice-cold PBS and lysed in radioimmunoprecipitation (RIPA) lysis buffer, and then, the protein of nuclear lysates was quantified by the BCA reagent, followed by western blot analysis.

### Immunofluorescence Assay

Immunofluorescence assay was performed as previously described (Chen et al., 2014). Briefly, J774 A.1 cells were seeded in a 6-well plate containing 1 × 10^6^ cells per well and cultured with or without ABPS (500 μg/ml) for 6 h. After that, cells were washed with ice-cold PBS, fixed in 3% paraformaldehyde solution, and permeabilized by 0.1% Triton X-100 in PBS. Cells were incubated with NF-κB-specific antibodies for 2 h, followed by incubated with conjugates of anti-rabbit IgG with FITC. Then, cells were incubated with PI and mounted for confocal microscopy.

### Statistical Analysis

All results were presented as mean ± S.D. Data were analyzed by one-way ANOVA using Student’s *t*-test. *P* values less than 0.05 were considered to be statistically significant.

## Results

### Effects of ABPS on the Release of Cytokines and NO in J774 A.1 Cells

**FIGURE 1:**
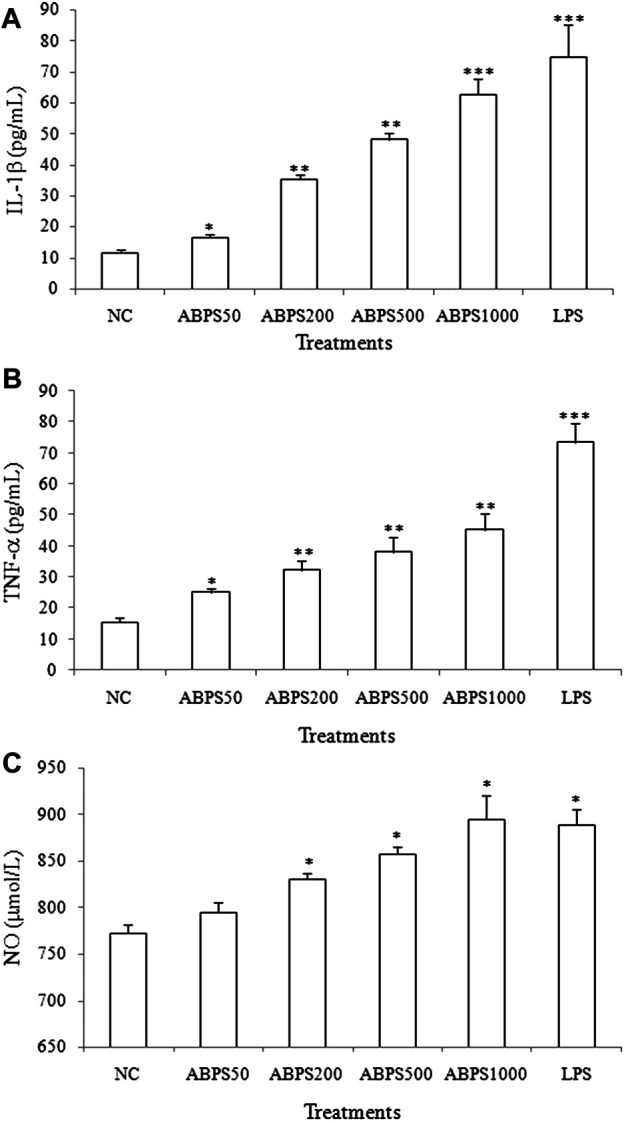
Effects of ABPS on the secretion of cytokines and NO in J774 A.1 cells. The cells were treated with ABPS (0, 50, 200, 500, and 1,000 μg/ml, respectively) or LPS (5 μg/ml) for 24 h. Cell culture medium was collected, and the secretion levels of cytokines **(A)** IL-1β, **(B)** TNF-α, and **(C)** NO were detected. NC: normal control; ABPS50: 50 μg/ml ABPS treated; ABPS200: 200 μg/ml ABPS treated; ABPS500: 500 μg/ml ABPS treated; ABPS1000: 1,000 μg/ml ABPS treated; and LPS: 5 μg/ml LPS treated (positive control). ^*^ *p* < 0.05, ^**^ *p* < 0.01, ^***^ *p* < 0.001 *vs.* NC. The values are presented as means ± SD.

To investigate the immunoregulatory activity of ABPS, we performed the experiment to detect the secretion of cytokines (IL-1β and TNF-α) induced by ABPS in J774 A.1 cells. As shown in Figure 1, the levels of IL-1β and TNF-α in ABPS-treated groups were significantly higher than those in control groups (NC). ABPS increased the secretion of IL-1β and TNF-α in a concentration-dependent manner, and the dose of 1,000 μg/ml presented the strongest stimulating effect on the cytokine production. LPS (5 μg/ml) significantly increased the level of IL-1β and TNF-α, compared with the NC group (*p* < 0.001). Reports showed that the secretion of cytokines accompanies oxidative bursts when cells were treated with foreign substances (Chen et al., 2014). To confirm the effect of ABPS on this event, the level of NO was measured. The levels of NO were significantly increased when the cells were treated with 200, 500, and 1,000 μg/ml ABPS or LPS (5 μg/ml) (*p* < 0.05), compared with the NC group. There was no significant difference between the level of NO in the 50 μg/ml ABPS-treated group and that in the NC group.

### Effects of ABPS on mRNA Expression of Cytokines in J774 A.1 Cells

**FIGURE 2:**
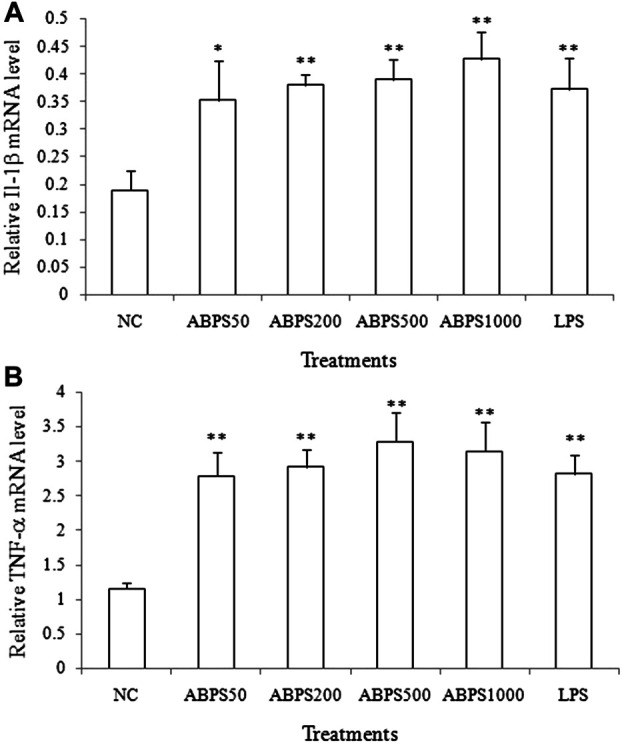
Effects of ABPS on the mRNA expression of IL-1β and TNF-α in J774 A.1 cells. The cells were treated with ABPS (0, 50, 200, 500, and 1,000 μg/ml, respectively) or LPS (5 μg/ml) for 24 h. Total RNA was isolated, and the mRNA expression levels of **(A)** IL-1β and **(B)** TNF-α were determined. NC: normal control; ABPS50: 50 μg/ml ABPS treated; ABPS200: 200 μg/ml ABPS treated; ABPS500: 500 μg/ml ABPS treated; ABPS1000: 1,000 μg/ml ABPS treated; and LPS: 5 μg/ml LPS treated (positive control). ^*^ *p* < 0.05, ^**^ *p* < 0.01, *vs.* NC. The values are presented as means ± SD.

To further confirm the stimulatory effect of ABPS on the secretion cytokines, the mRNA expressions of IL-1β and TNF-α were investigated. As shown in Figure 2, the mRNA expressions of IL-1β and TNF-α in ABPS (50, 200, 500, and 1,000 μg/ml) treatment groups were markedly increased, compared with the NC group (*p* < 0.05, *p* < 0.01). LPS (5 μg/ml) significantly increased the expression of IL-1β and TNF-α mRNA, compared with the NC group (*p* < 0.01). This result suggested that ABPS upregulated the mRNA expression of cytokines in J774 A.1 cells.

### TLR4-Dependent Activation of Macrophages by ABPS

**FIGURE 3:**
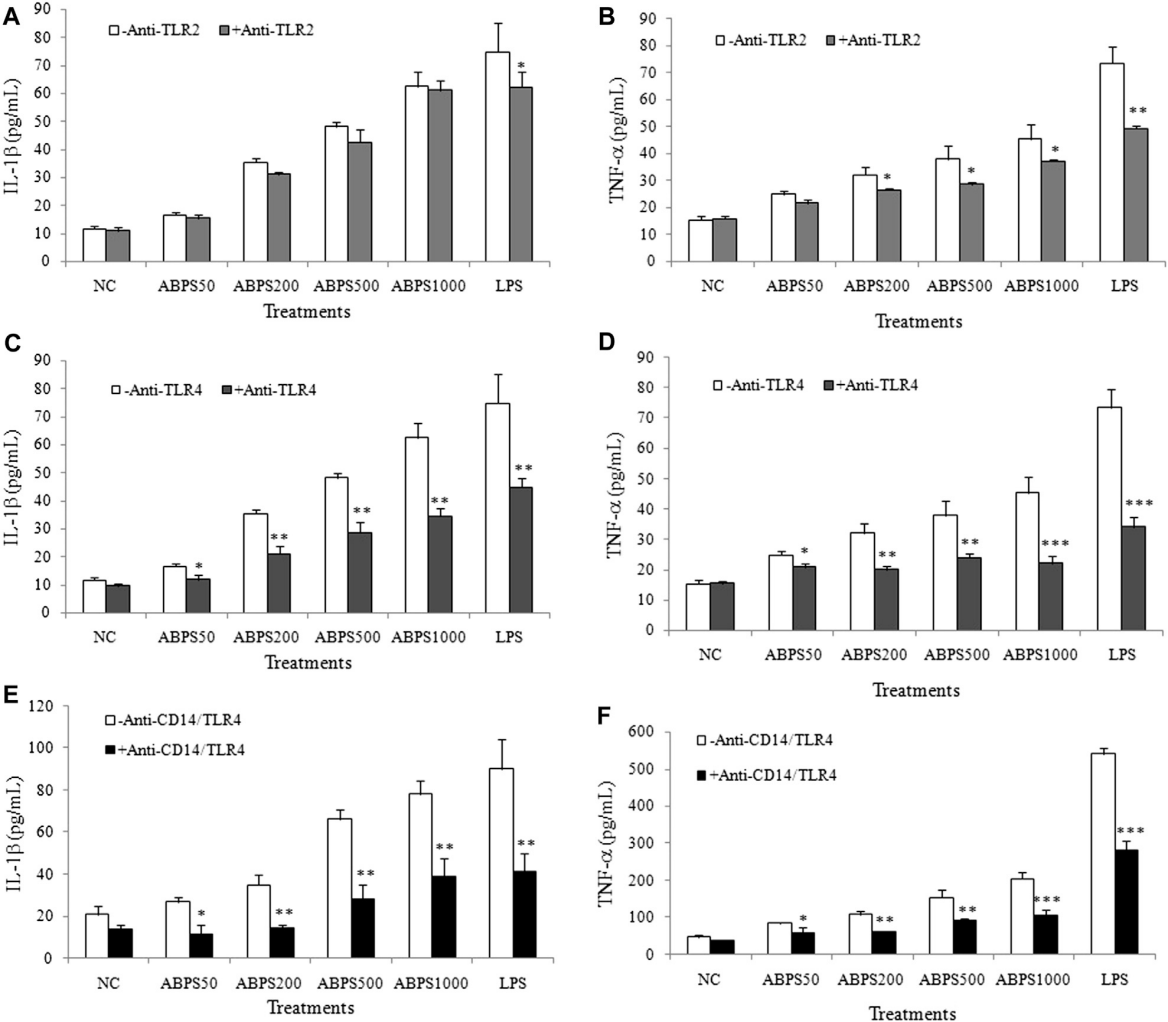
ABPS activation of macrophages depends on TLR4. **(A)** IL-1β and **(B)** TNF-α. The cells were pretreated with or without 20 μg/ml anti-TLR2 antibody for 1 h and then treated with ABPS (0, 50, 200, 500, and 1,000 μg/ml, respectively) or LPS (5 μg/ml) for 24 h. Cell culture medium was collected, and the secretion levels of cytokines were detected. **(C)** IL-1β and **(D)** TNF-α. Cells were pretreated with or without 20 μg/ml anti-TLR4 antibody. **(E)** IL-1β and **(F)** TNF-α. Cells were pretreated with or without 20 μg/ml anti-CD14/TLR4 antibody. NC: normal control; ABPS50: 50 μg/ml ABPS treated; ABPS200: 200 μg/ml ABPS treated; ABPS500: 500 μg/ml ABPS treated; ABPS1000: 1,000 μg/ml ABPS treated; and LPS: 5 μg/ml LPS treated (positive control). ^*^ *p* < 0.05, ^**^ *p* < 0.01, ^***^ *p* < 0.001 *vs.* control (without antibody treatment group). The values are presented as means ± SD.

The secretion of cytokines (IL-1β and TNF-α) can be mediated by various signals, in which Toll-like receptors (TLRs) are the most important ones. Most studies have shown that polysaccharides recognize and bind to TLRs, which initiate the immune response (Yin et al., 2019). For further insight into the mechanism of the secretion of cytokines by ABPS, J774 A.1 cells were incubated with ABPS after pretreatment with TLR antagonist (anti-TLR2, anti-TLR4, and anti-CD14/TLR4 antibody), and then, the secretion of IL-1β and TNF-α in the supernatant was detected. As shown in Figure 3A, the level of IL-1β was not significantly different between anti-TLR2 pretreatment and without anti-TLR2 pretreatment in all ABPS-treated groups and the NC group. Anti-TLR2 can significantly reduce the level of IL-1β induced by LPS (*p* < 0.05). However, in ABPS-treated groups, the levels of IL-1β were significantly decreased with anti-TLR4 pretreatment, compared with those without anti-TLR4 pretreatment. In the NC groups, with or without anti-TLR4 pretreatment did not affect the secretion of IL-1β (Figure 3C). The levels of IL-1β in ABPS-treated groups were significantly decreased with anti-CD14/TLR4 pretreatment, compared with those without anti-CD14/TLR4 pretreatment. In the NC groups, anti-CD14/TLR4 did not affect the secretion of IL-1β (Figure 3E).

In 200, 500, and 1,000 μg/ml ABPS treatment groups, the secretions of TNF-α were significantly decreased when the cells were pretreated with anti-TLR2 (*p* < 0.05), compared with those without anti-TLR2 treatment. In 50 μg/ml ABPS and NC groups, with or without anti-TLR2 pretreatment did not change the expression of TNF-α (Figure 3B). In ABPS-treated groups, the levels of TNF-α were significantly decreased with anti-TLR4 pretreatment, compared with those without anti-TLR4 pretreatment. In the NC groups, with or without anti-TLR4 pretreatment did not affect the secretion of TNF-α (Figure 3D). The levels of TNF-α in ABPS-treated groups with anti-CD14/TLR4 antibody were significantly decreased, compared with those without anti-CD14/TLR4 pretreatment (Figure 3F). The antagonist (anti-TLR2, anti-TLR4, and anti-CD14/TLR4 antibody) pretreatment significantly decreases the expression of IL-1β and TNF-α induced by LPS (5 μg/ml).

**FIGURE 4:**
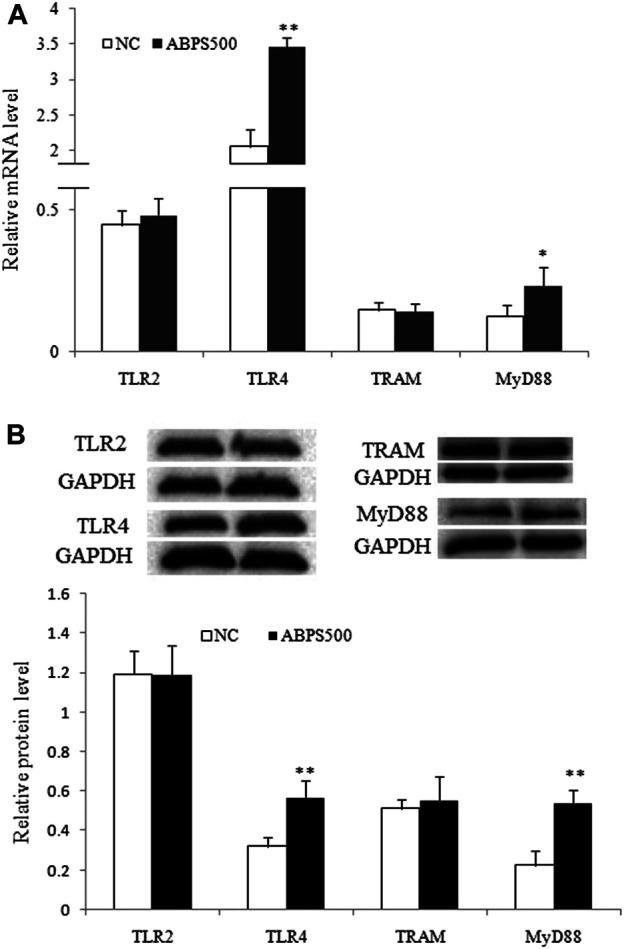
ABPS increased the expression of TLR4 and MyD88 in J774 A.1 cells. **(A)** The mRNA expression of TLR2, TLR4, TRAM, and MyD88. **(B)** The protein expression of TLR2, TLR4, TRAM, and MyD88. The blot shown is representative of one of the three similar experiments. Cells were treated 500 μg/ml ABPS for 24 h. NC: normal control; ABPS500: 500 μg/ml ABPS treated. ^*^ *p* < 0.05, ^**^ *p* < 0.01 *vs.* NC. The values are presented as means ± SD.

Furthermore, the mRNA and protein level of TLR4 were significantly increased in the ABPS treatment group (*p* < 0.01), compared with that in the control group (Figure 4). ABPS treatment did not change the expression of mRNA and the protein of TLR2. These results suggested that the activation of macrophages by ABPS was mainly through TLR4.

### ABPS Activated TLR4 With the Selection of the MyD88-dependent Pathway

TLR4 signaling can be modulated by MyD88-dependent or MyD88-independent pathways (Mokhtari et al., 2021). To confirm the pathway activated by ABPS, we detected the expression of two key molecules, TRAM and MyD88, which are, respectively, located in the downstream of TLR4. As shown in Figure 4, ABPS had no effect on the expressions of mRNA and the protein of TRAM. However, the expressions of mRNA and the protein of MyD88 were significantly increased by ABPS treatment. These results indicated that ABPS activated TLR4 with the selection of the MyD88-dependent downstream pathway.

### ABPS Promotes the Expression and Nuclear Translocation of NF-κB

**FIGURE 5:**
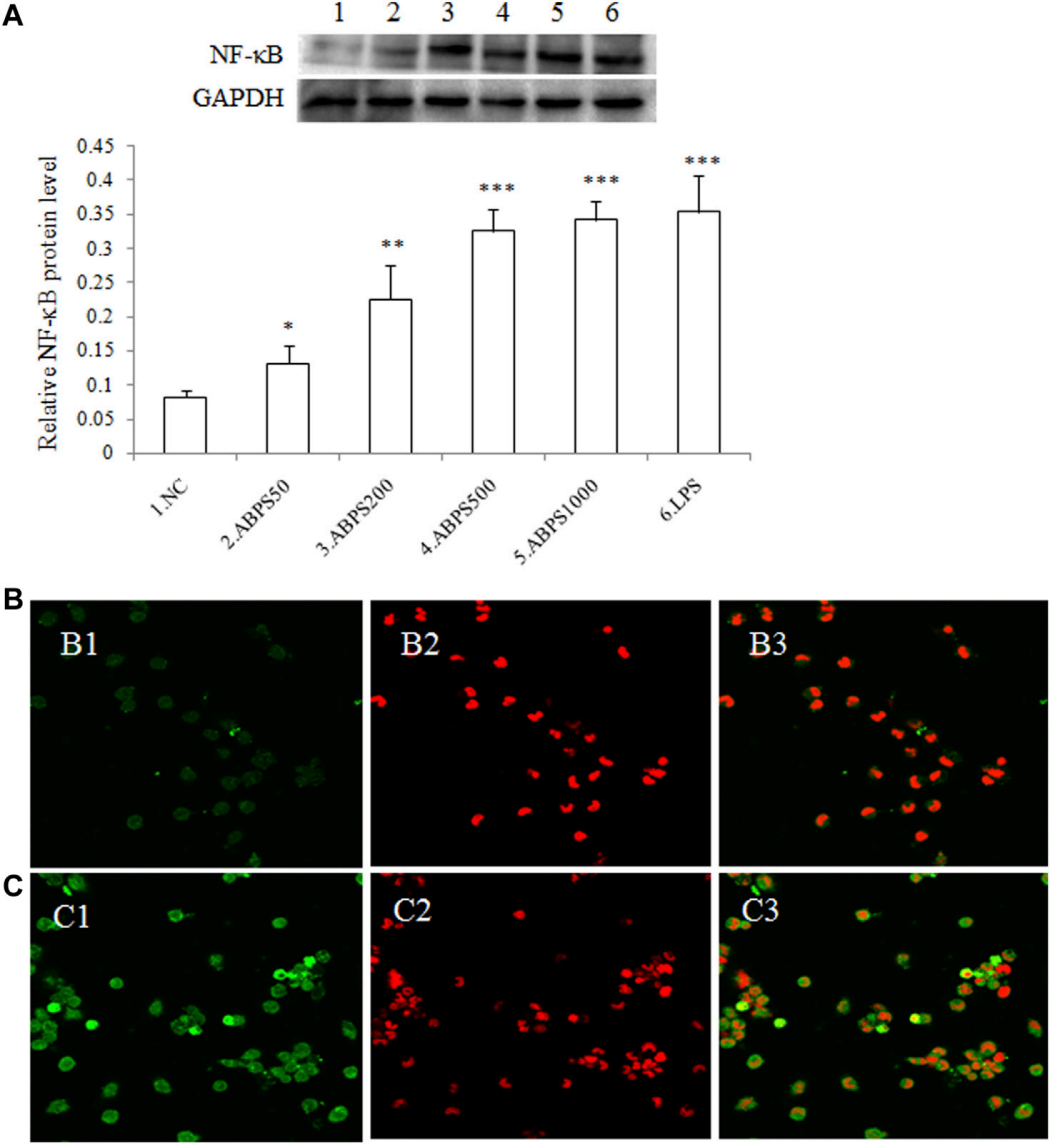
Effect of ABPS on the expression and nuclear translocation of NF-κB in J774 A.1 cells. **(A)** The expression of NF-κB in J774 A.1. Cells were treated with ABPS (0, 50, 200, 500, and 1,000 μg/ml, respectively) or LPS (5 μg/ml) for 24 h. Nuclear extracts were prepared and determined by western blot. NC: normal control; ABPS50: 50 μg/ml ABPS treated; ABPS200: 200 μg/ml ABPS treated; ABPS500: 500 μg/ml ABPS treated; ABPS1000: 1,000 μg/ml ABPS treated; and LPS: 5 μg/ml LPS treated (positive control). ^*^ *p* < 0.05, ^**^ *p* < 0.01, ^***^ *p* < 0.001 *vs.* NC. The values are presented as means ± SD. **(B)** and **(C)** ABPS promoted nuclear translocation of NF-κB. Cells were stained with FITC-tagged antibody (B1: control; C1: 500 μg/ml ABPS) or propidium iodide (PI) (B2: control; C2: 500 μg/ml ABPS). Micrographs (B3: control; C3: 500 μg/ml ABPS) represent merged images obtained through the red and green fluorescence channels.

It is known that stimulation of TLR4 will mediate downstream signaling cascades that ultimately lead to the activation and nuclear translocation of nuclear factor-kappa B (NF-κB) (El-Zayat et al., 2019) and result in the secretion of cytokines (Santoni et al., 2015). To determine the activation of NF-κB by ABPS, western blot (WB) and laser scanning confocal microscopy (CLSM) were used to examine the nuclear translocation of NF-κB induced by ABPS. As shown in Figure 5A, the levels of NF-κB in the nucleus were increased markedly by ABPS in a dose-dependent manner. Furthermore, immunofluorescence staining revealed that ABPS markedly promoted the expression and nuclear translocation of NF-κB (Figures 5B,C). NF-κB mainly existed in the cytoplasm in the control group (Figure 4B), and the staining was more intense in the nucleus by ABPS treatment (Figure 5C).

### Effect of PDTC on ABPS-Induced Cytokine Secretion

**FIGURE 6:**
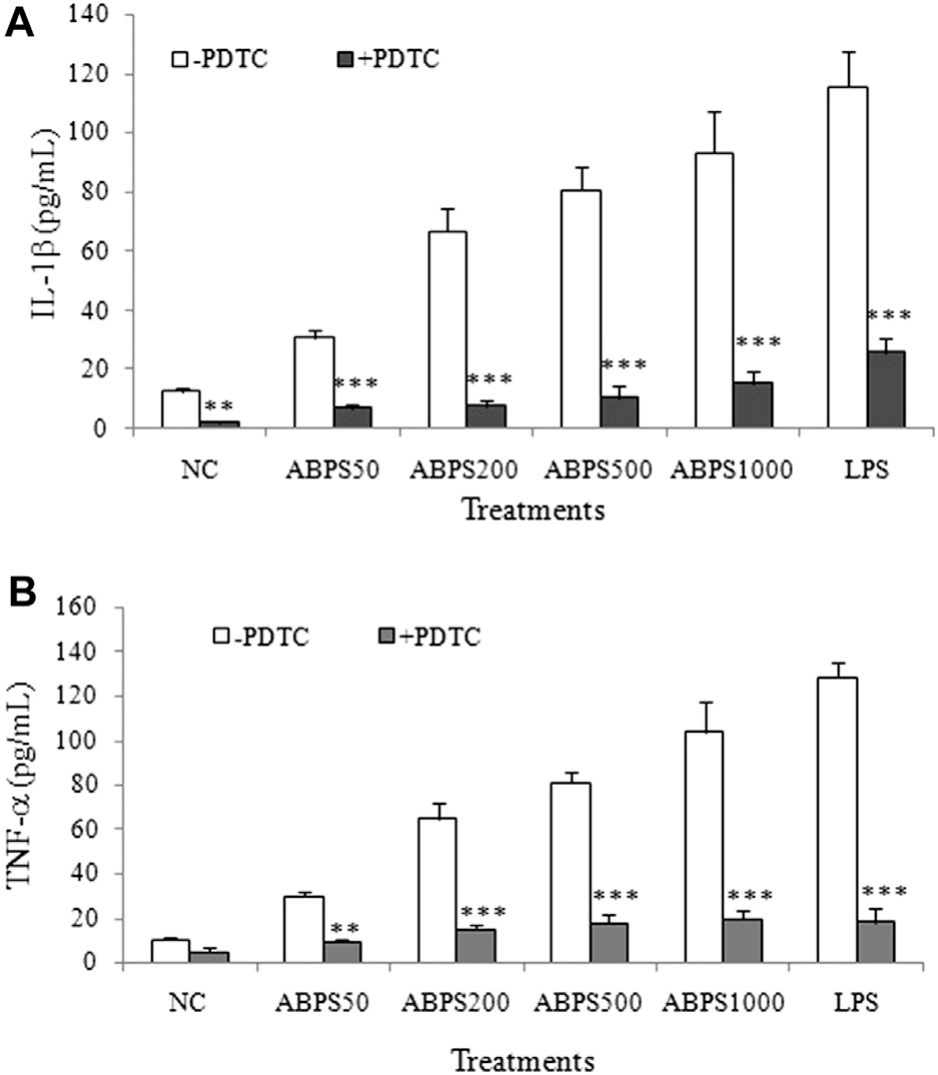
Effect of NF-κB inhibitor (PDTC) on ABPS induced the secretion of IL-1β and TNF-α. The cells were pretreated with or without 100 μg/ml anti-TLR2 antibody for 2 h and then treated with ABPS (0, 50, 200, 500, and 1,000 μg/ml, respectively) or LPS (5 μg/ml) for 24 h. Cell culture medium was collected, and the secretion levels of cytokines **(A)** IL-1β and **(B)** TNF-α were detected. NC: normal control; ABPS50: 50 μg/ml ABPS treated; ABPS200: 200 μg/ml ABPS treated; ABPS500: 500 μg/ml ABPS treated; ABPS1000: 1,000 μg/ml ABPS treated; and LPS: 5 μg/ml LPS treated (positive control). ^**^ *p* < 0.01, ^***^ *p* < 0.001 *vs.* control (without antibody treatment group). The values are presented as means ± SD.

As described previously, NF-κB activation can regulate specific genes that encode a set of proteins such as proinflammatory cytokines (IL-1β and TNF-α) (Moresco et al., 2011). To further confirm the NF-κB-dependent macrophage activation of ABPS, the effects of ABPS on J774 A.1 cells after pretreatment with PDTC, a specific inhibitor of NF-κB, were investigated. As shown in Figure 6, ABPS significantly increased the secretion of IL-1β and TNF-α, and these increases can be almost completely abolished by PDTC. The result suggests that the activation of macrophages by ABPS is mediated by the NF-κB signal pathway.

## Discussion

Fructans are sucrose-derived compounds and represent an important class of polysaccharide in plants (Dobrange et al., 2019; Young et al., 2021). Based on structural characteristics, the fructans can be categorized as follows: inulin (β-2,1-linked fructose residues), levan (β-2,6-linked fructose residues), and graminan (β-2,1-linked and β-2,6-linked fructose residues) (Dobrange et al., 2019). ABPS, containing both β-2,1-linked and β-2,6-linked fructose residues, possesses diverse biological activities including inhibiting osteoclast formation and bone absorption, antitumor, and immunomodulatory (Lin et al., 2021). However, the underlying mechanism of the immunomodulatory activity of ABPS is not well understood. In the present study, we attempted to unveil intracellular signaling pathways in response to the activation of macrophages by ABPS.

Fructans have been demonstrated to exert immunomodulatory effects, which were attributed to the ability to enhance the production of nitric oxide (NO) and other immunostimulatory factors (e.g., IL-1, IL-6, IL-10, interferon-gamma (IFN)-γ, and TNF-α) (Dobrange et al., 2019). Therefore, we first investigated the effect of ABPS in J774 A.1 cells by manifesting the production of cytokines (IL-1β and TNF-α) and NO. To investigate the dose–effect relationship of ABPS, the doses of 50, 200, 500, and 1,000 μg/ml were used in the experiment. The high concentration of ABPS (500 and 1,000 μg/ml) was used to generate a strong immune response. In the present study, ABPS can promote the secretion of IL-1β and TNF-α in a dose-dependent manner. Meanwhile, the mRNA expression levels of IL-1β and TNF-α were markedly increased with ABPS treatment, suggesting that ABPS stimulated macrophages to secrete cytokines by regulating the gene expression. Activated macrophages release NO, an intracellular messenger molecule, which mediates a variety of biological functions including immune responses (Gong et al., 2017). ABPS markedly promotes the NO production in macrophages. These results suggest that ABPS promotes immune activity by stimulating the release of cytokines and NO.

IL-1β and TNF-α are released by multiple signaling transduction pathways, including TLRs, which are widely expressed on the surface of macrophages (Roeder et al., 2004; Wang et al., 2021). TLRs are the most important ones of PRRs, which form the cornerstone of the innate immune response (El-Zayat et al., 2019). Among the members of TLRs, TLR2 and TLR4 can bind polysaccharide ligands, activating the downstream signaling axes (Wang et al., 2021). CD14, mainly produced by monocytes and macrophages, recognizes and binds various structures and subsequently transfers them to TLRs (Allam et al., 2015). Inulin, the most common type of fructans, was shown to possess direct signaling capacity on human immune cells by activating primarily TLR2 (Vogt et al., 2013), and peptidoglycan recognition protein 3 (PGlyRP3) signaling has also been proposed (Zenhom et al., 2011). The β-2,6 fructans as well as other polysaccharides have shown immunomodulatory activities; however, the exact mechanisms underpinning the immunomodulatory activities remain elusive, and one potential underpinning mechanism for microbial levan specifically is through the interaction with TLR4 and C-type lectin receptors (CLRs) (Young et al., 2021). This prompted us to investigate the effects of ABPS on TLR2, TLR4, and CD14, and anti-TLR2, anti-TLR4, and anti-CD14/TLR4 antibodies were used to block the signaling. The results showed that the blockade of TLR2 signaling has no significant effect on the secretion of cytokines induced by ABPS. However, the blockade of TLR4 (or CD14/TLR4) signaling by antagonistic antibody significantly reduced the secretion of cytokines induced by ABPS, suggesting that ABPS mainly activates TLR4, which triggers the downstream signaling. Meanwhile, ABPS promotes the mRNA and protein expression of TLR4 and has no effect on the expression of TLR2. These results indicate that the immune response induced by ABPS is mainly through TLR4.

TLR4 signaling can be modulated by MyD88-dependent or MyD88-independent pathways (Mokhtari et al., 2021). Upon ligand binding to TLR4, the adaptor protein, primarily MyD88, is recruited. MyD88 is further associated with IL-1R-associated kinase (IRAK) family kinases and results in the formation of a “myddosome” complex, which initiates various responses, and triggering the downstream pathway (Ahmed et al., 2021; Saikh, 2021). Our results showed that ABPS can significantly increase the mRNA and protein expression of MyD88. However, ABPS has no effect on the mRNA and protein expression of TRAM, which mediates TLR4 signaling in a MyD88-independent/TRIF-dependent way (McGettrick et al., 2006), indicating that ABPS activates TLR4 signaling *via* the MyD88-dependent pathway.

The TLR intracellular signals ultimately lead to the activation and nuclear translocation of the transcription factors such as NF-κB and IRFs (El-Zayat et al., 2019), which dictate the consequences of the innate immune response activation (Ahmed et al., 2021). The nuclear translocation of NF-κB regulates the expression of a variety of genes, associated with the innate and adaptive immune responses (Hayden and Ghosh, 2011). Our results showed that ABPS promoted the expression and nuclear translocation of NF-κB in a dose-dependent manner. The nuclear translocation of NF-κB by ABPS was further confirmed by CLSM. To further confirm the role of NF-κB in the secretion of cytokines induced by ABPS, PDTC (an NF-κB inhibitor) was used to block the NF-κB signaling. It was observed that the blockade of NF-κB signaling by PDTC significantly reduced the secretion of cytokines (IL-1β and TNF-α) induced by ABPS, suggesting that ABPS induced signal transduction of macrophage activation via NF-κB signaling.

In summary, this study used a macrophage model to investigate the immunomodulatory effect of ABPS and the underlying mechanism. Taken together, the results present in this study suggest that ABPS induces macrophage activation through the TLR4/MyD88/NF-κB signaling pathway. This study also provides a basis for the development and utilization of ABPS as an immunostimulant or agonist of TLR4.

## Data Availability

The original contributions presented in the study are included in the article/Supplementary Material; further inquiries can be directed to the corresponding author.
